# Dislocation force of scleral flange-fixated intraocular lens haptics

**DOI:** 10.1186/s12886-024-03369-x

**Published:** 2024-03-05

**Authors:** Spela Stunf Pukl, Martin Kronschläger, Manuel Ruiss, Stéphane Blouin, Emre Rüştü Akcan, Oliver Findl

**Affiliations:** 1 Vienna Institute for Research in Ocular Surgery (VIROS), A Karl Landsteiner Institute, Hanusch Hospital, Heinrich-Collin Str. 30, AT-1140 Vienna, Austria; 2 Medical Faculty, University of Ljubljana, Ljubljana, Slovenia; 3 Eye Hospital, University Clinical Centre Ljubljana, Ljubljana, Slovenia; 4 Ludwig Boltzmann Institute of Osteology at the Hanusch Hospital of OEGK and AUVA Trauma Centre Meidling, Medical Department Hanusch Hospital, Vienna, Austria

**Keywords:** Scleral fixation, Flange-fixated IOL, Yamane technique, Dislocation force

## Abstract

**Purpose:**

To measure the dislocation forces in relation to haptic material, flange size and needle used.

**Setting:**

Hanusch Hospital, Vienna, Austria.

**Design:**

Laboratory Investigation.

**Methods, main outcome measures:**

30 G (gauge) thin wall and 27 G standard needles were used for a 2 mm tangential scleral tunnel in combination with different PVDF (polyvinylidene fluoride) and PMMA (polymethylmethacrylate haptics). Flanges were created by heating 1 mm of the haptic end, non-forceps assisted in PVDF and forceps assisted in PMMA haptics. The dislocation force was measured in non-preserved cadaver sclera using a tensiometer device.

**Results:**

PVDF flanges achieved were of a mushroom-like shape and PMMA flanges were of a conic shape. For 30 G needle tunnels the dislocation forces for PVDF and PMMA haptic flanges were 1.58 ± 0.68 N (*n* = 10) and 0.70 ± 0.14 N (*n* = 9) (*p* = 0.003) respectively. For 27 G needle tunnels the dislocation forces for PVDF and PMMA haptic flanges were 0.31 ± 0.35 N (*n* = 3) and 0.0 N (*n* = 4), respectively. The flange size correlated with the occurring dislocation force in experiments with 30 G needle tunnels (*r* = 0.92), when flanges were bigger than 384 micrometres.

**Conclusions:**

The highest dislocation forces were found for PVDF haptic flanges and their characteristic mushroom-like shape for 30 G thin wall needle scleral tunnels. Forceps assisted flange creation in PMMA haptics did not compensate the disadvantage of PMMA haptics with their characteristic conic shape flange.

## Background

The flanged intra-scleral IOL (intraocular lens) fixation technique includes formation of two scleral tunnels, externalisation of the IOL haptics, and haptic end heating to create a flange that seals the scleral tunnel [1]. 

When performed appropriately, this technique has been shown to offer stable IOL positioning in eyes without capsular support, however the surgical steps are challenged and are, along with the equipment and materials, still a matter of several modifications [2, 3]. 

The most important risk after the surgery is erosion of the flange out of the scleral tunnel and through the conjunctiva. On the contrary, during the surgery and immediately postoperatively, the meticulous surgical steps can be complicated by flange slippage trough the scleral tunnel into the eye. The stability of the IOL during the procedure and immediately after the surgery mostly depends on fixing force of the flange - its resistance to slip through the scleral tunnel.

Sclera is the opaque, firm collagenous outermost layer of the eye. Its stiffness and stress-stain properties differ at various regions of the eye, and they do change significantly depending on the healing processes [4–7]. Scleral tissue has a non-linear behaviour with low stiffness at lower stress, and increasing stiffness at higher stress. Its in-*vivo* resistance thus depends also on the IOP changes [7]. 

Before the onset of scleral tunnel healing and fibrosis, and especially in cases of intra- or early post-operative lover IOP, the fixing force of the flanges depends exclusively on the relationship of the scleral tunnel dimensions versus the flange size and shape.

Typically, the scleral tunnel in Yamane technique is positioned 2 mm behind the limbus, is angled slightly posteriorly, and is parallel to the limbus.

Relationship between the diameter of the tunnel, which is initially determined by the needle thickness, versus the haptic and flange size, change after the surgery. However, during and immediately after the surgery, a suitable size relationship would add additional safety and stability of the flanges and IOL.

The present study was designed to measure the forces needed to dislocate haptic flanges of known dimension out of characteristic scleral tunnels in human autopsy eye sclera in order to better understand the potential impact of size matching of needle to haptic and flange dimensions for scleral fixation.

## Methods

Four different 3-piece IOLs, that are often used for the scleral fixation technique, were included in this experimental study. The haptics were of two different materials: Polyvinylidene fluoride– PVDF (*n* = 10): CT Lucia 202 (Carl Zeiss Meditec AG, Jena, Germany) and the Avansee Preset (Kowa, Tokyo, Japan), and Polymethylmethacrylate– PMMA (*n* = 9): Sensar AR40 IOL (Johnson&Johnson, Santa Ana, USA) and Tecnis ZA9003 IOL (Johnson&Johnson, Santa Ana, USA).

A typical tangential 2 mm long scleral tunnel was created with a 30 gauge (G) thin-wall needle with a length of 13 mm (TSK Laboratory Europe, Oisterwijk, Netherlands). For comparison of the fixing forces, a 27 gauge (G) standard needle, with a length of 12 mm (Sterican B. Braun, Hessen, Germany) was used alternatively.

Flanging of the introduced haptic was done with a low-temperature cautery from the MST Scleral IOL Fixation Solutions Pack (MicroSurgical Technology, Redmond, USA). Previously studied techniques for flanging were used as follows: (i) for the IOL with PMMA, the haptic was grasped with forceps 1 mm from the tip during flange formation; (ii) for the IOL with PVDF haptics a non-grasping technique was used. The flange was wedged into the outer ostia of the scleral tunnel.

Experiments were carried on non-preserved human scleral tissue provided by the Vienna Eye Bank.

**Fig. 1:**
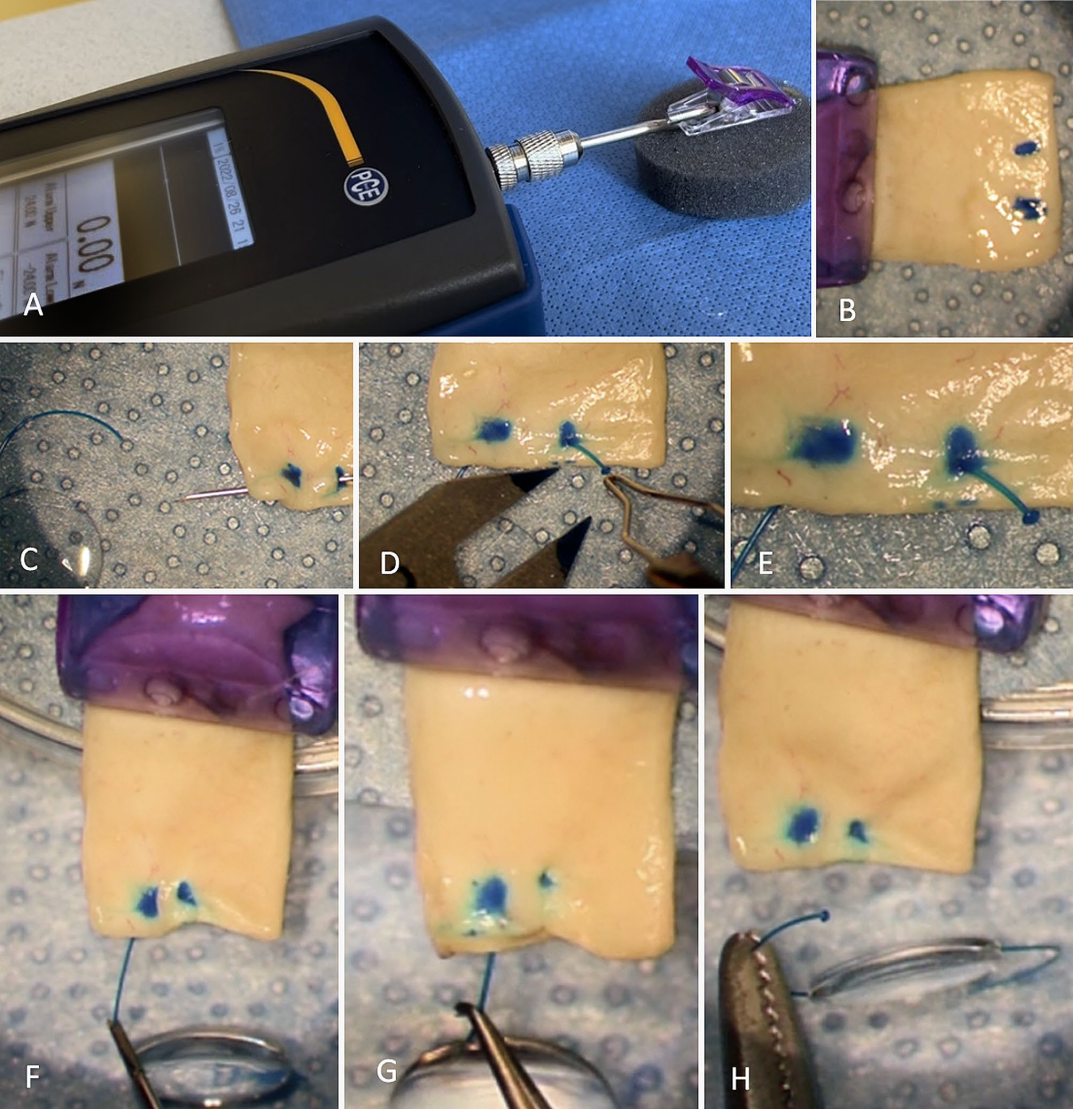
Measurement of dislocation force: **A**: An electronic force gauge instrument was secured in the horizontal plane, **B**: scleral tissue fixated into a straight clamp, **C**: 30 G thin wall needle used for a 2 mm scleral tunnel, **D**: 1 mm haptic flanging with no-grasp technique, **E**: the flange, **F** to **H**: pulling of the haptic until dislocated

For measurement of the dislocation force of the intra-scleral flange fixated IOL haptic a bench setup was designed. An electronic force gauge instrument (PCE-DFG-N20, PCE Instruments) was secured in the horizontal plane. A straight clamp was attached to the instrument. The clamp was applied on the sclera parallel to the scleral tunnel. The haptic exiting the scleral tunnel at its interior was pulled with forceps while measuring the applied force. The maximal force until the flange slipped though was recorded. The thickness of the pulled-out flange was then analysed with light microscopy and measured (Fig. 1).

For flange analysis and measurement, the haptics of the IOLs with dislocated flanges were observed in reflection mode by circular differential interference contrast using an optical upright microscope (Axio Scope.A1, Carl Zeiss Meditec AG, Jena, Germany) equipped with a 5x EC Epiplan Neofluar high-density differential interference contrast lens (Carl Zeiss Meditec AG). A compact charge-coupled device monochrome camera (Lumenera Corp.) recorded 1392 × 1040 pixels images with a resolution of 1.3 mm/pixels. Microscopic images of each haptic and flange were taken. The diameter of the peripheral end of each haptic and each flange were measured perpendicularly to the long axis of the haptic with the aid of the straight tool in ImageJ.

For statistical analysis the SPSS software (IBM, USA) was used, the groups were compared by the Mann-Whitney U test, the diameter of flanges as well as the material of the haptics and size of the needle were correlated by the ANOVA test to the dislocation force measured. For correlation analysis the Pearson correlation coefficient was used.

## Results

The mean dislocation force for PVDF haptic flanges from the 2 mm tangential scleral tunnel created with a 30 G thin wall needle was 1.58 ± 0.68 N (*n* = 10) and the mean dislocation force for PMMA haptic flanges under the same settings was 0.70 ± 0.14 N (*n* = 9). The difference was statistically significant, *p* = 0.003, Mann-Whitney U test (Fig. 1).

**Fig. 2:**
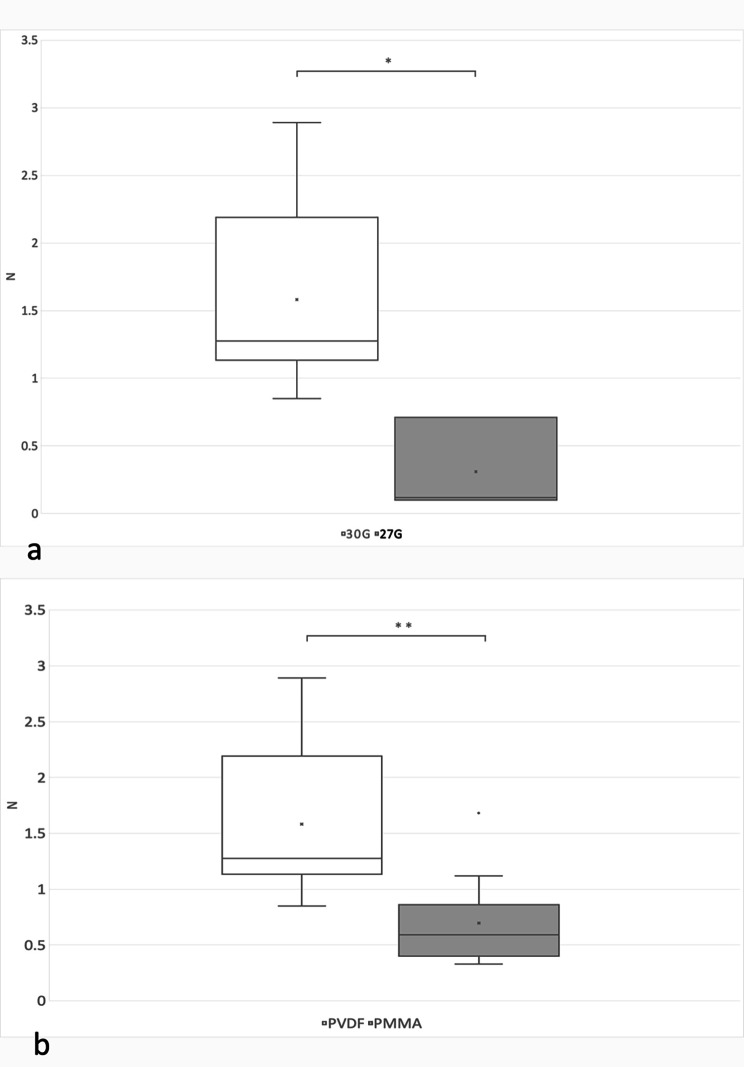
(**a**) Dislocation forces measured in Newtons (N), 2 mm tangential scleral tunnel created with a 30 G thin wall needle or 27 G standard needle, measurements for flanges of PVDF haptics. * *p* < 0.05. (**b**) Dislocation forces measured in Newtons (N), 2 mm scleral tunnel, 30 G thin wall needle and 3-piece IOLs with haptics of different materials: PVDF, PMMA. ** *p* < 0.01

When the 2 mm long tangential tunnel was for comparison to the 30 G created with a 27 G standard needle, the flange slippage occurred in experiments with PMMA haptics. The dislocation forces could be measured only in few experiments with the PVDF haptics (*n* = 3) and were substantially and statistically significantly lower compared to experiments with 30 G needles and PVDF haptics (Fig. 2a): PVDF group, 0.31 ± 0.35 N (*p* = 0.011, Fig. 2b).

**Fig. 3:**
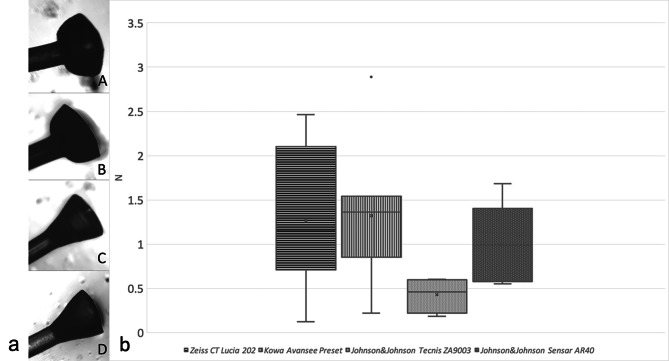
(**a**) Microscopic images of flanges of different IOLs: **A**– Zeiss CT Lucia 202, **B**– Kowa Avansee Preset, **C**– Johnson&Johnson Tecnis ZA9003, **D**– Johnson&Johnson Sensar AR40. (**b**) Dislocation force in Newtons for different IOLs, 30 G thin wall needle tunnel (Zeiss CT Lucia 202: *n* = 6, Kowa Avansee Preset: *n* = 5, Johnson&Johnson Tecnis ZA9003: *n* = 5, Johnson&Johnson Sensar AR40: *n* = 4)

Mean values of dislocation force and standard deviations when the tunnel was made with a thin wall 30 G technique, as well as sizes and characteristic shapes of flanges for different IOLs are in Table 1; Fig. 3a and b.

PVDF flanges were of mushroom like shape and PMMA flanges were of conic like shape (Table 1; Fig. 3a).

**Table 1:** Dislocation force and flange size for different IOLs

IOL label	IOL company	Mean size of the flange, micrometres (µm)		Dislocation force, Newtons (N)	
		Mean	SD	Mean	SD
Zeiss CT Lucia 202	Carl Zeiss Meditec (Jena, Germany)	376.40	26.9	1.31	1.0
Kowa Avansee Preset	Kowa (Tokyo, Japan)	360.63	13.8	1.37	1.0
Johnson&Johnson Tecnis ZA9003	Johnson&Johnson, Abbott Medical Optics (Santa Ana, USA)	356.30	18.2	0.46	0.1
Johnson&Johnson Sensar AR40	Johnson&Johnson, Abbott Medical Optics (Santa Ana, USA)	409.05	10.4	0.99	0.5

* Scleral tunnel 2 mm, 30 G thin wall needle, heating 1 mm of haptic, non-forceps assisted in PVDF haptics and forceps assisted in PMMA haptics

### Flanges

**Fig. 4:**
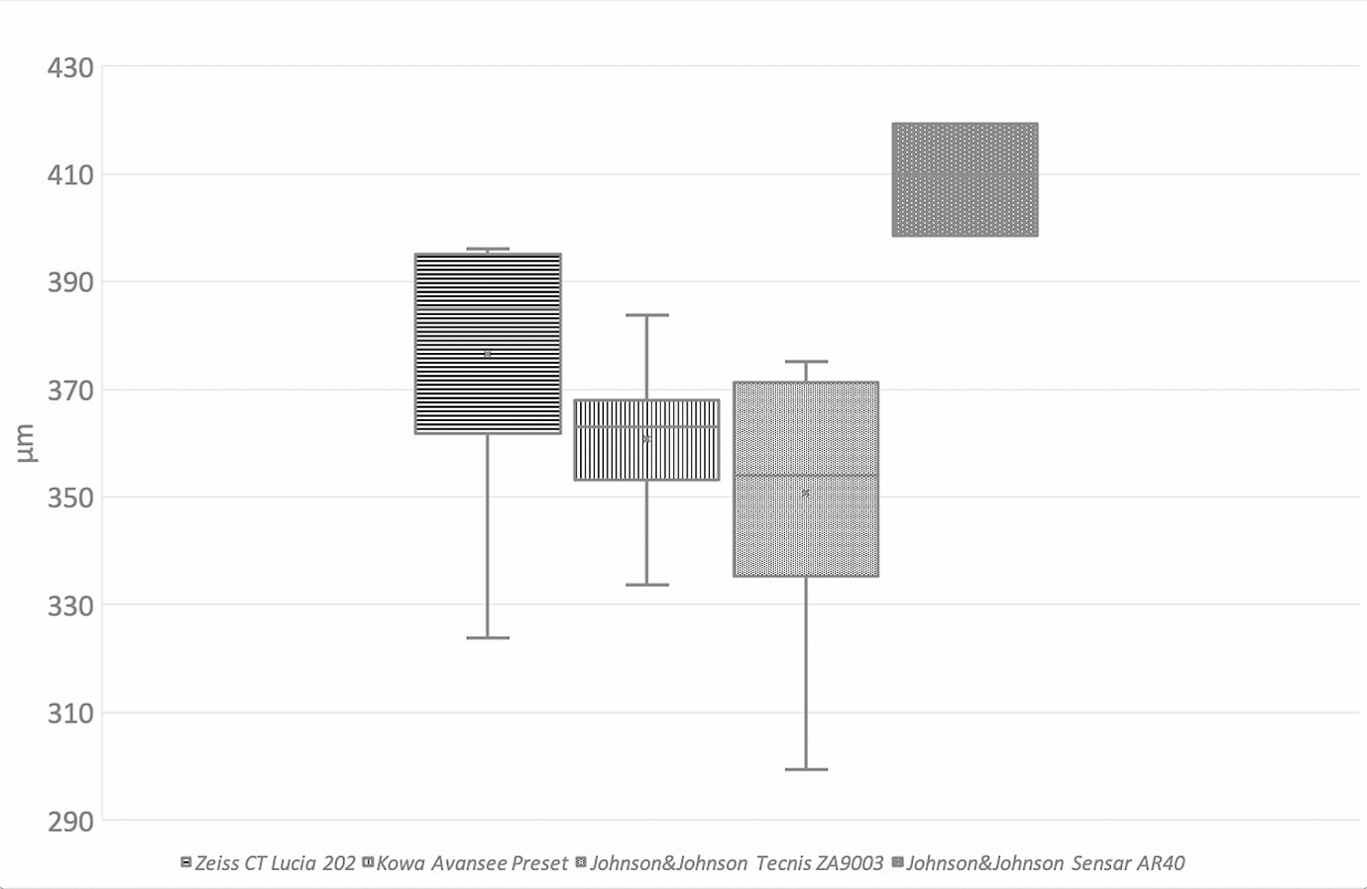
Flange size in micrometres for all analysed flanges (Zeiss CT Lucia 202: *n* = 6, Kowa Avansee Preset: *n* = 9, Johnson&Johnson Tecnis ZA9003: *n* = 10, Johnson&Johnson Sensar AR40: *n* = 3)

Mean flange diameter of all analysed flanges (flanges that slipped through the tunnel and those that produced resistance) was 376 ± 27 μm for the Zeiss CT Lucia 202 IOLs (Figs. 4), 361 ± 14 μm for the in Kowa Avansee Preset IOLs, 356 ± 18 μm for the Johnson&Johnson Tecnis ZA903 IOLs, and 409 ± 11 μm for the Johnson&Johnson Sensar AR40 IOLs. All of the flanges from PVDF haptics were created without grasping the haptic, and result in a mushroom-like shape (Fig. 3a). On the other hand, flanges from PMMA haptics were performed by flanging 1 mm from the haptic end and grasping the haptic 1 mm from the haptic tip. They were of a conic-like shape (Table 1; Fig. 3a).

**Fig. 5:**
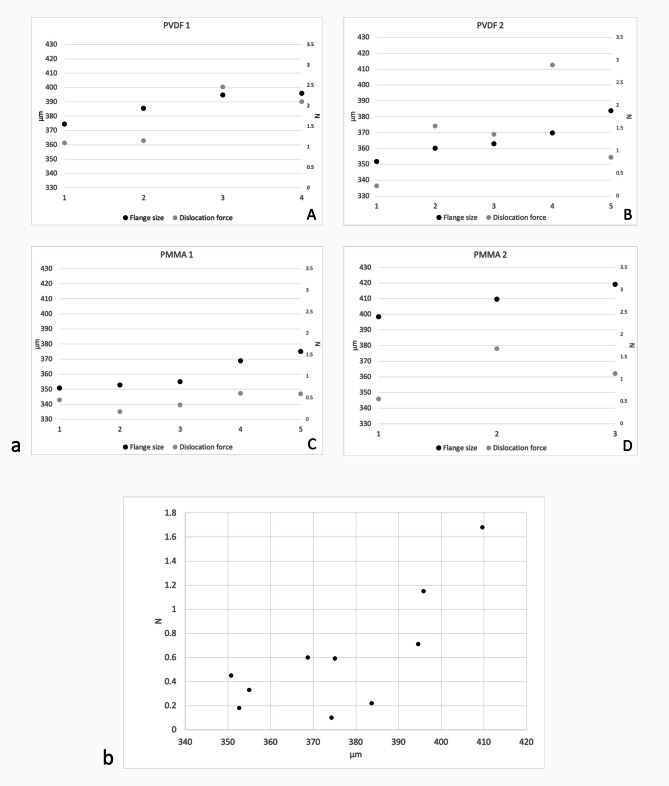
(**a**) Flange size in micrometres (left of double y axis) and dislocation force in Newtons (right of double y axis) for **A**: PVDF1 (Zeiss CT Lucia 202) *n* = 4; **B**: PVDF2 (Kowa Avansee Preset) *n* = 5; **C**: PMMA1 (Johnson&Johnson Tecnis ZA9004) *n* = 5; and **D**: PMMA2 (Johnson&Johnson Sensar AR40) *n* = 3. (**b**) Flange size in micrometres (x axis) and dislocation force in Newtons (y axis) for all flanges, dislocated out for 30 G thin wall tunnels, which produced resistance

In 30 G thin-wall needle tunnels, a higher dislocation force was measured in larger flanges in for most of the performed experimental dislocations (Fig. 5a: A-D). With all the IOLs, the correlation of size to force is a flat curve for all IOLs up to the flange size of 384 micrometres (correlation coefficient– 0.04). For flanges larger than 385 microns, flange size correlated with dislocation force (0.92) (Fig. 5b).

In a 27 G needle tunnel no dislocation force could be obtained with PMMA haptic flanges since they all slipped through the tunnel and the mean dislocation force for PVDF haptic flanges was 0.31 ± 0.347 N, *n* = 3 (Fig. 2a).

## Discussion

The scleral fixation technique with flanged intrascleral haptic fixation, first described by Yamane, has been an important addition to the techniques used for fixating IOLs in eyes without capsular support [1]. Standardisation of the technique, however, may be needed for increasing safety, such as avoiding either haptic dislocation or oversized flanges which may cause protrusion of haptic ends under and through the conjunctiva. The numerous combinations of different haptic materials and thicknesses of IOLs available, as well as the needles used for externalisation, make standardisation challenging.

According to the experimental study with cadaver sclera, the flange is an effective way to stabilize the haptic in the immediate post-implantation period [8]. Ma et al. further showed that the haptic material and flange to tunnel size coefficient do influence the dislocation of flanged haptic from cadaveric sclera [9]. 

Incorporating the knowledge of how to obtain an optimal flange [10], and imitating the surgical steps, as described by Yamane concerning the scleral tunnel preparation [1, 2], the present laboratory study was performed to measure dislocation forces immediately after the haptic insertion and flanging.

The dislocation forces for PVDF haptic flanges were significantly higher, meaning more stable IOL, than for the PMMA haptic flanges. Among the PVDF haptic flanges, substantially higher dislocation forces were measured when a 30 G thin wall needle was used for creation of the 2 mm long tangential scleral tunnel as compared to the 27 G needle tunnel. The findings are in accordance with previously published results from Ma et al. However, in their study the scleral tunnel was not tangential and 2 mm long as described in the original technique report [1], and also the PMMA haptics were flanged without the forceps holding technique, which was proved to results in a broader flange [9]. We thus propose the scleral tunnel in Yamane style scleral fixation to be performed as originally described − 2 mm behind the limbus, angled slightly posteriorly, parallel to the limbus - with a 30 G thin wall needle, 2 mm tangentially trough the sclera in order to achieve the optimal flange fixation force.

As far as flanges, the flange shape depends on the haptic material and flanging technique [10, 11] and can, if not consistent, result in instability of the flange in the scleral tunnel, or on the other hand conjunctival erosions [12]. The flange shape of PVDF haptics appears to be independent of whether forceps are used for assistance while heating. Conversely, with PMMA haptics, a forceps gripping is needed to attain mushroom-like or a prominent cone-like shaped flange, which was proposed to provide maximum hold in the scleral tunnel and minimum leak to the sub-conjunctival space [10]. Not using the forceps in PMMA haptics during heating, the flanges creates less prominent conic flanges [10]. Forceps assisted flange creation in PMMA haptics during the experiments reported here however, did not compensate the disadvantage of PMMA haptic characteristic conic shaped flange, resulting in lower dislocation forces, which means less stability of the IOLs with PMMA haptics in the scleral tunnel. Appropriate type of IOL selection is thus also an important step. A PVDF haptic IOL represents a more secure option for Yamane scleral fixation according to measurements in the present study.

Ma et al. performed a no-grasp haptic heating technique and reported flange sizes of 0.5–2.0 mm. They found that heating the haptic beyond 1.0 mm did not result in a larger or differently shaped flange [9]. Dislocation forces in their experiments correlated to the flange to needle diameter ratio, speaking in favour of sufficiently large flanges. The optimal shape of a prominent cone with PMMA haptics may be obtained with forceps-assisted heating [10], which however in the contrary to our expectations, did not prove higher dislocation forces in PMMA haptic IOLs compared to the published data [9]. 

A mushroom-like shape of the PVDF haptics resulted in a better stability in our study. Even though the flange sizes were comparable to the flanges of the PMMA haptics (Table 1), the dislocation forces were larger for PVDF haptics (Table 1). However, a grasp assisted prominent conic shape of the PMMA haptic according to intuitive thinking offers easier slip of the flange into the scleral tunnel and a good seal immediately after burial of the flange into the scleral tunnel. While mid-term flange stability, reported in the literature [13], results also from the fibrotic sclera response. Further studies are needed to assess the sealing properties of differently shaped flanges.

Heating 1 mm of the haptic end in theory results in a flange diameter of approximately 350 μm [10]. The flanges performed showed a slight variability in flange size (Table 1). The largest diameters were measured for the Sensar AR40 IOLs, which does, however, have the thickest haptics compared to the other three IOLs used [14]. The flange should thus be performed by heating at least 1 mm of the haptic end in order not to be smaller than 350 μm.

Being of the same importance as the flange, a precise sclera tunnel formation has several important impacts during and after the procedure. When a 30 G thin wall needle was used for the scleral tunnel, a larger flange size when more than 380 micrometres in diameter positively correlated to a higher dislocation force regardless of the IOL type. This suggest that independent of the haptic material, a well-formed flange will provide enough stability of the IOL in a 30 G thin wall needle tunnel [10], which is long and deep in sclera enough. However, beyond the stability of the IOL, safety after the surgery in terms of postoperative leak and endophthalmitis risk depends also on long enough scleral tunnel (at least 2 mm) and sufficient flange burial [12]. To large flange diameter might not burry and seal and could represent a risk for flange erosions, postoperative leakage, invasion of microorganisms [12]. The length and orientation of the scleral tunnel additionally stabilise the flanged haptic. The close-to-limbus tangential insertion [7], long scleral tunnel (at least 2 mm), and appropriate burial of the flange into the sclera covered with tenon’s capsule and conjunctiva provide stability, and appear to also be important to avoid flange erosions, act as an effective barrier to invasion of microorganisms, which may otherwise result in postoperative endophthalmitis [12].

The scleral tunnel length may result in instability of the IOL if too short resulting in slippage of the flange through the sclera post-operatively resulting in subluxation of the IOL, or resulting in IOL decentration if unpaired to the contralateral tunnel length, During the experiments, the non-preserved cadaver sclera tissue elasticity change, if used beyond the first two days after retrieval, was noticed. This speaks for early processes of tissue degradation and/or hydration. Tissue quality is of course an important aspect when interpreting these laboratory results concerning dislocation forces. Additionally, the clinical and long-term stability of flanged intrascleral fixation of three-piece IOL haptics also depend on the elastic and healing/scarring properties of the sclera. Measurements of the dislocation forces in this cadaver scleral model thus only applies to the specific aspect of the procedure and long-term clinical follow-up studies are needed.

## Conclusions

The present experimental study was performed to imitate the intraoperative and early postoperative stability of flanged intrascleral fixation of three-piece IOL haptics in an aphakic eye. Using the model of non-preserved cadaveric sclera, the 2 mm tangential tunnel provided highest dislocation forces if created with a 30 G thin wall needle and when a PVDF haptic flange was used, suggesting that the scleral tunnel diameter, length, and direction, as well as proper IOL type selection are essential for the fixation force in Yamane scleral fixation. The post-dislocation analysis of flanges proved that a larger flange diameter resulted in higher dislocation forces independent to the haptic material when larger than 384 microns. Forceps assisted flange creation with PMMA haptics did not compensate for the disadvantage of the less stable PMMA characteristic conic-shaped flange. However, flanging of 1 mm haptic end assures a sealing flange not smaller than 350 microns.

## Data Availability

The datasets analysed during the current study are available from the corresponding author on reasonable request.

## Abbreviations

G: Gauge
IOL: Intraocular lens
PMMA: Polymethylmethacrylate
PVDF: Polyvinylidene fluoride
